# Mononuclear Phagocyte Activation Is Associated With the Immunopathology of Psoriasis

**DOI:** 10.3389/fimmu.2020.00478

**Published:** 2020-03-25

**Authors:** Mariana C. Costa, Camilla S. Paixão, Débora L. Viana, Bruno de O. Rocha, Maíra Saldanha, Lícia M. H. da Mota, Paulo R. L. Machado, Carla Pagliari, Maria de Fátima de Oliveira, Sergio Arruda, Edgar M. Carvalho, Lucas P. Carvalho

**Affiliations:** ^1^Laboratório de Pesquisas Clínicas, LAPEC, Instituto Gonçalo Moniz, Salvador, Brazil; ^2^Ciências Médicas, Universidade de Brasília (UnB), Brasilia, Brazil; ^3^Serviço de Imunologia, Hospital Universitário Professor Edgard Santos, Universidade Federal da Bahia, Salvador, Brazil; ^4^Departamento de Patologia, Universidade de São Paulo (USP), São Paulo, Brazil; ^5^Laboratório Avançado de Saúde Pública (LASP), Instituto Gonçalo Moniz, Salvador, Brazil; ^6^Instituto Nacional de Ciência e Tecnologia em Doenças Tropicais, INCT-DT, Salvador, Brazil

**Keywords:** psoriasis, mononuclear phagocytes, cytokines, sCD14, sCD163

## Abstract

Psoriasis is a chronic, inflammatory disease affecting the skin and joints. The pathogenesis of this disease is associated with genetic, environmental and immunological factors, especially unbalanced T cell activation and improper keratinocyte differentiation. Psoriatic lesion infiltrate is composed of monocytes and T cells, and most studies have focused on the participation of T cells in the pathogenesis of this disease. Here we investigated the contribution of mononuclear phagocytes in the immunopathology observed in psoriatic patients. Significant increases in the levels of TNF, IL-1β, CXCL9, as well as the soluble forms of CD14 and CD163, were observed within the lesions of psoriatic patients compared to skin biopsies obtained from healthy individuals. Moreover, we found an association between the levels of CCL2, a monocyte attractant chemokine, and disease severity. In conclusion, our findings suggest a potential role for mononuclear phagocytes in the pathogenesis of psoriasis.

## Introduction

Psoriasis is an autoimmune skin disorder that affects ~2% of the world's population, and is characterized by the exacerbated proliferation/activation of keratinocytes (1). Most studies designed to elucidate the pathogenesis of psoriasis have documented the notable participation of T cells, particularly Th1, in the development of psoriatic skin lesions (2). In this context, IFN-γ production has been found to be increased within psoriatic lesions (2). TNF, a cytokine produced by a variety of cells, including T cells, NK cells and mononuclear phagocytes, is also known to participate in the pathogenesis of psoriasis by promoting the infiltration of inflammatory cells, as well as inducing the production of proinflammatory cytokines through the activation of the transcriptional factor NFκB (3). More recently, it was documented that IL-23, produced by dendritic cells, activates Th17 cells to produce IL-17A, IL-17F and IL-22 (4, 5). These cytokines are known to contribute to neutrophil recruitment and directly activate keratinocytes, thereby promoting hyperplasia in these cells (4–6).

Less attention has been paid to the contribution of mononuclear phagocytes in the immunopathology observed in psoriatic individuals. Human circulating monocytes constitute a heterogeneous population of cells with distinct phenotypical and functional features. Based on surface CD14 and CD16 expression, these cells can be subdivided into classical (CD14++CD16–), intermediate (CD14++CD16+) and non-classical (CD14+CD16+) subsets (7, 8). High frequencies of intermediate monocytes producing TNF and IL-1β have been associated with immunopathology and documented in inflammatory diseases, such as rheumatoid arthritis and cutaneous leishmaniasis (9, 10).

To investigate the contribution of mononuclear phagocytes to the pathogenesis of psoriasis, we determined the frequency of monocyte subsets and markers of mononuclear phagocyte activation at lesion sites in psoriatic patients. Although no alterations in the frequencies of monocyte subsets were detected in these patients, we found increased levels of mononuclear phagocyte activation markers within psoriatic lesions, and established a positive correlation between levels of the monocyte attractant CCL2 and severity of disease.

## Methods

### Patients

The present cross-sectional study involved 26 patients with active psoriasis vulgaris and 16 healthy subjects (HS), all aged 18 years or older. The patients either had no history of systemic treatment, or had not underwent phototherapy or systemic therapy for no less than the minimum time necessary to completely eliminate the previously used drug. This study received approval from the Institutional Review Board of the School of Medicine of the Federal University of Bahia (FMB-UFBA), and all subjects provided written informed consent.

### ELISA

Serum and biopsies were collected from each study subject. Biopsies were extracted from lesions using a 4mm punch and placed in sterile medium containing 1 ml of RPMI-1640 (Gibco Laboratories, Grand Island, NY, USA) supplemented with 10% fetal bovine serum (Gibco Laboratories, Grand Island, NY, USA), 10 IU/ml penicillin and 100 μg/ml streptomycin. Culturing was carried out for 48 h at 37°C under 5% CO_2_. Biopsy and serum supernatants were collected and stored at −70°C. The levels of TNF, IL-1β, sCD14, sCD163, CCL2, and CXCL9 were determined by ELISA (R&D Systems, Minneapolis, MN, USA) according to the manufacturer's instructions. Results are expressed in pg/ml.

### Flow Cytometry

To determine the frequency of cells expressing CD14 and CD16, peripheral blood mononuclear cells (PBMC) (500,000 cells) were obtained from heparinized blood and stained with anti-MHC II, anti-CD14 and anti-CD16 antibodies (BD biosciences, San Jose, CA, USA) for 20 min at 4°C. Cells were then fixed with 2% paraformaldehyde and acquired on a FACScanto II cell counter (BD bioscience, San Jose, CA, USA) (200,000 events/sample). To determine the cell frequency in skin biopsies, a punch (4 mm) biopsy was performed in healthy skin and in psoriatic lesions. Tissue biopsies were incubated with Liberase TL (200 μg/ml) (Roche Diagnostics, Germany) for 1 h at 37°C. They were then macerated and filtered with a 40 μm BD (Falcon cell strainer, BD Pharmingen). Cells were stained with anti-MHC II, anti-CD14 and anti-CD16 antibodies (BD biosciences, San Jose, CA, USA), as described above and acquired on a FACScanto II cell counter (BD bioscience, San Jose, CA, USA).

### Immunohistochemistry

Tissues obtained from 5 psoriatic skin and 5 controls, fixed in buffered formaldehyde and embedded in paraffin. Deparaffinization and rehydration of 5-μm thick sections were performed using xylene and alcohol PA and antigen retrieval, using citrate buffer pH 6.0 at 96°C for 20 min. Immunohistochemistry reactions were performed after blockage of peroxidase activity with 3% hydrogen peroxide for 10 min and proteins with Protein Block Serum-Free (Dako) for 15 min. The slides were incubated overnight at 4°C with Monoclonal Mouse TNF (Cell Signaling Technology). Mouse and Rabbit Peroxidase Kit/Horseradish Peroxidase KP500 (Diagnostic BioSystems) were used to perform the reaction according to the manufacturer's recommendations. For double staining, slides were incubated overnight at 4°C with rabbit anti-TNF antibody (Biorbyt), orb18766, dilution 1:200, and developed in black color with nickel diaminobenzidine. Slides were then incubated for 1 h with mouse anti-CD68 monoclonal antibody, (Dako) M0876, dilution 1:100, at 37°C, and developed in green color with PermaGreen/HRP K074, (BioSystems). A polimer Polink HRP (GBI Labs) was used to perform the reaction according to the manufacturer's recommendations. Finally, sections were dehydrated and mounted with Permount (Thermo Fisher Scientific) and glass coverslips.

### Statistical Analysis

Mann-Whitney testing was used to compare HS and psoriasis groups, while non-parametric (Spearman's) correlation analysis was used to evaluate correlations. *P* < 0.05 was considered statistically significant, and all tests were two-tailed.

## Results

**Figure 1:**
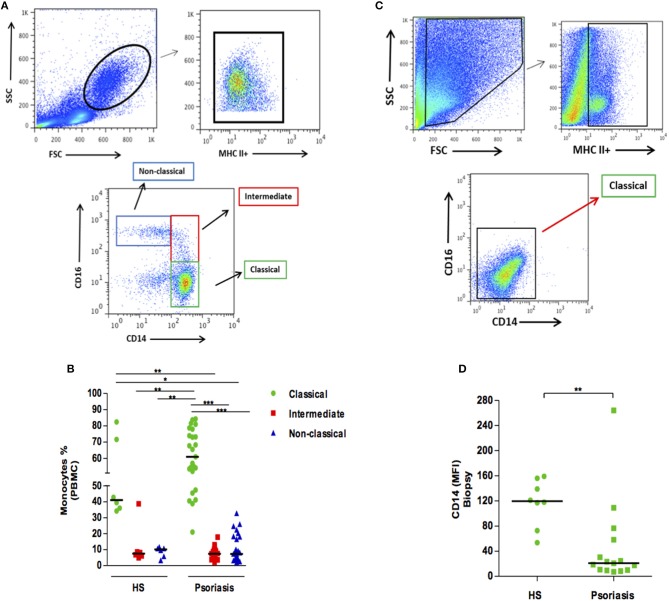
Frequency of monocyte subsets in patients with psoriasis vulgaris and healthy individuals. Skin biopsies (4 mm) and PBMCs were obtained, and *ex-vivo* staining for MHC II, CD14, and CD16 was performed. **(A)** Representative plots of monocyte subsets in PBMCs of a patient with psoriasis. **(B)** Frequency of circulating monocyte subsets in healthy subjects (HS) (*n* = 6) and patients with psoriasis (*n* = 25). **(C)** Representative plots of monocyte subsets in a lesion biopsy from a patient with psoriasis. **(D)** Mean fluorescent intensity (MFI) of CD14 in skin biopsies from HS (*n* = 8) and in lesions from patients with psoriasis (*n* = 23). Statistical comparisons were performed using the Kruskal-Wallis test and Dunn's post-test *p ≤ 0.05, **p ≤ 0.01, ***p ≤ 0.001.

The frequency of intermediate monocytes (CD14+, CD16+) is increased in the blood of patients with inflammatory diseases, such as rheumatoid arthritis or leishmaniasis (9–11). Here we did not find significant differences in the frequencies of circulating monocyte subsets when comparing psoriatic and healthy individuals (Figures 1A,B). Since conditions in the peripheral blood environment may differ from sites of inflammation, we then determined mononuclear phagocyte populations at lesion sites and compared these to healthy skin. We found that mononuclear phagocytes within the skin expressed lower levels of CD14 and CD16, both in psoriatic patients and HS. However, the intensity of CD14 expression was found to be lower in psoriatic lesions, suggesting the increased activation of macrophages in psoriatic lesions (Figures 1C,D).

**Figure 2:**
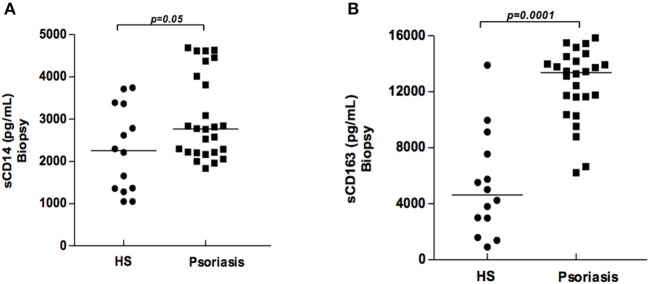
Production of sCD14 and sCD163 in supernatants of biopsy cultures of lesion (psoriatic patients) and skin (healthy subjects). Biopsy fragments (4 mm) were obtained from lesions of psoriatic patients (*N* = 26) and skin from healthy subjects (HS) (*N* = 14), and cultured for 48 h. sCD14 **(A)** and sCD163 **(B)** concentrations were determined by ELISA (results presented in pg/ml). The Mann-Whitney test was used to compare medians between groups.

The classical activation of mononuclear phagocytes induces the cleavage of CD14 and CD163 molecules, as well as the release of their soluble forms. Higher levels of sCD14 and sCD163 were found in the supernatants of biopsy cultures from patients with psoriasis in comparison with HS (Figures 2A,B). However, no correlation between sCD14 and sCD163 with disease severity (PASI) was found. This finding indicates that mononuclear phagocytes are activated at lesion sites, and suggests the participation of these cells in the inflammatory response.

**Figure 3:**
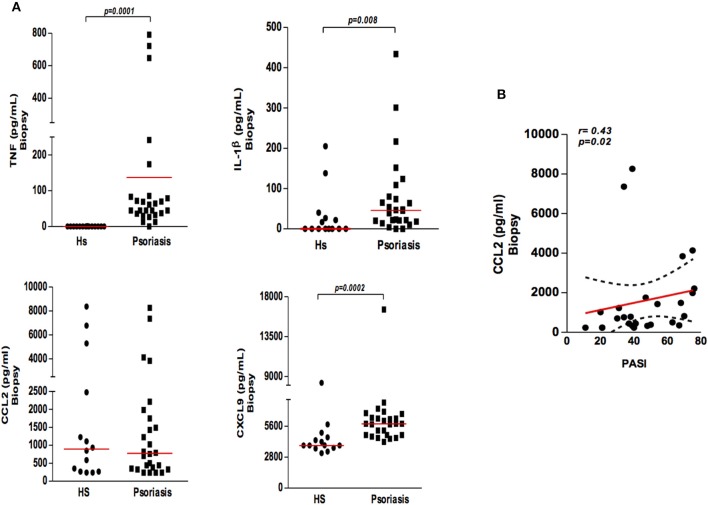
Production of cytokines and chemokines in the supernatants of biopsy cultures of lesion (psoriatic patients) and skin (healthy subjects). **(A)** Biopsies (4 mm) were obtained from lesions of psoriatic patients (*N* = 26) and skin from healthy subjects (HS) (*N* = 14) and cultured for 48 h. Cytokine and chemokine (TNF, IL-1β, CXCL9, and CCL2) concentrations were determined by ELISA (results presented in pg/ml). The Mann-Whitney test was used to compare medians between groups. **(B)** Correlation between CCL2 levels in cultured supernatants from patients with psoriasis and disease severity (PASI). Data were analyzed using Spearman's correlation testing.

To investigate the contribution of mononuclear phagocytes in the inflammatory process occurring in psoriatic lesions, we quantified the cytokines and chemokines mainly secreted by mononuclear phagocytes in biopsy cultures from psoriasis patients and skin from HS. We found that cultures from biopsies obtained from psoriatic lesions had higher levels of TNF, IL-1β, and CXCL9 than those from HS skin cultures (Figure 3A). Interestingly, although no differences in CCL2 levels were detected between psoriatic patients and HS, a positive correlation between CCL2 and severity of disease was documented (Figure 3B). Altogether, our data suggest activation of mononuclear phagocytes in psoriatic lesions and the secretion of inflammatory mediators.

**Figure 4:**
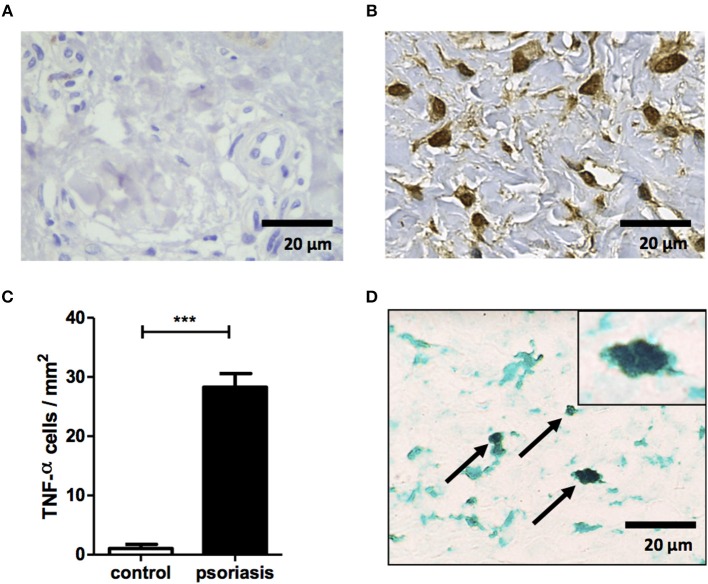
Mononuclear phagocytes contribute to TNF production within psoriatic lesion. Immunostaining for TNF in biopsy of healthy subjects (*n* = 5) and psoriasis patients (*n* = 5). **(A,B)** Representative immunostaining from healthy subject **(A)** and psoriasis lesion **(B)**. **(C)** Mean ± SD from 5 individuals from each group. **(D)** Representative double staining for CD68 (green) and TNF (black) in a psoriatic lesion. Slides were photographed (ten randomized fields from each section). Original magnification x40 and x100 (top right corner **D**). Scale bar = 20 μm. ***P < 0.001.

Our data show the presence of mononuclear phagocytes activation specific molecules, sCD14 and sCD163, in lesion of psoriasis patients. In order to investigate the contribution of mononuclear phagocytes to inflammation within psoriatic lesions, we performed immunohistochemistry for TNF. As expected, psoriatic skin had increased TNF production compared with skin from HS (Figures 4A–C). Mononuclear phagocytes (CD68+) were important source of this cytokine (Figure 4D). Altogether, our data documents the participation of mononuclear phagocytes in inflammation of psoriatic lesions.

## Discussion

Psoriasis is a chronic autoimmune disease in which T cells are known to play an important role in its pathogenesis. The participation of macrophages in the pathogenesis of psoriasis has been previously suggested with a key role for TNF and IL-1β (12, 13). In a mouse model of psoriasis, depletion of macrophages led to a decrease in severity of the disease (14). Here we investigated the contribution of mononuclear phagocytes in the inflammatory response present in lesions of psoriatic patients. Additionally, we found that activated macrophages within the lesions of these patients produce inflammatory mediators, which possibly contributes to the immunopathology of this disease.

Mononuclear phagocytes can infiltrate sites of inflammation and exacerbate immunopathology, as observed in tuberculosis, leishmaniasis and rheumatoid arthritis (9–11, 15, 16). The shedding of molecules CD14 and CD163 occurs upon macrophage activation. The soluble form of CD14 has been used as a biomarker to predict pulmonary exacerbation in cystic fibrosis, and is increased in patients with chronic hepatitis (17, 18). In addition, high levels of circulating sCD163 have been documented in a variety of chronic infections and autoimmune disorders (19–22). Here we detected lower expression of CD14 on the surface of macrophages from lesions in psoriatic patients when compared to HS, as well as higher levels of the soluble forms of CD14 and CD163, suggesting the participation of these cells in the pathogenesis of this disease. The cleavage of CD14 can be induced by a variety of stimuli, including TLR ligands and cytokine signaling, e.g., IL-6 and IL-1β (23). Interestingly, the endocytosis of sCD14 triggers the production of TNF and IL-1β by macrophages through the activation of NFκB and the inflammasome, leading to an inflammatory feedback loop (24).

To confirm the activation of mononuclear phagocytes and the participation of these cells in T cell recruitment, we measured levels of IL-1β, TNF, and CXCL9 in psoriatic lesions and found high levels of these inflammatory mediators. TNF and IL-1β, potent inflammatory molecules primarily secreted by macrophages and dendritic cells, induce inflammation through vascular endothelium activation and the induction of pro-inflammatory cytokine production (25). The mechanism(s) by which mononuclear phagocytes become activated in psoriasis is not known. One hypothesis is that IL-17, a cytokine commonly observed in psoriatic lesions, induces inflammatory cytokine production by macrophages through the activation of MAPKs, NFkB and AP-1 (26). Another hypothesis is that sCD14 activates NFkB molecules and inflammasomes (24). The data available in the literature reporting on the elevated IL-1β levels in psoriasis lesions, which is consistent with our findings, as well as significantly decreased levels of this cytokine in response to anti-inflammatory therapy (27, 28), support the notion that psoriasis patients may benefit from the use of drugs that downregulate IL-1β production.

CXCL9 is an important chemokine involved in T cell recruitment, and CCL2 is known to recruit monocytes, memory T cells and dendritic cells to sites of inflammation. These soluble factors are primarily produced by macrophages and participate in the pathogenesis of various diseases, such as autoimmune, autoinflammatory, metabolic, infectious, and neurodegenerative diseases, thereby contributing to the severity of the pathological process (29–33). Although higher levels of CXCL9 were present in psoriatic lesions than in HS skin, no associations with disease severity were observed; by contrast, CCL2 levels were found to be positively correlated with disease severity.

Finally, to confirm the participation of mononuclear phagocyte in the inflammation of psoriatic lesion we detected TNF-producing mononuclear cells within lesions of psoriasis patients. Mononuclear phagocytes may contribute to inflammation in many ways, including induction of NFκB expression, IL-8 and IL-17 production (34–37). In a previous report, in a set of five patients with pustular psoriasis, depletion of CD14+CD16+ circulating monocytes ameliorated symptoms and, interestingly, no change in TNF was observed, what suggests that other factors produced by monocytes may play a role in the pathogenesis of the disease (38). Pustular psoriasis is a more inflammatory presentation of the disease where, besides mononuclear phagocytes, neutrophils plays important role in inflammation (39). However, it has been documented that TNF contribute to vulgar psoriasis presentation, considering that targeting TNF using biologics improves symptoms (40). Taken together, the results herein present convincing evidence of the deleterious role played by mononuclear phagocytes in the exacerbation of immunopathology, and suggest that the inhibition of the soluble products secreted by these cells may benefit psoriatic patients.

## Data Availability Statement

All datasets generated for this study are included in the article/supplementary material.

## Ethics Statement

The studies involving human participants were reviewed and approved by the Institutional Review Board of the School of Medicine of the Federal University of Bahia. The patients/participants provided their written informed consent to participate in this study.

## Author Contributions

MC, EC, and LC: design and concept. CPai, DV, MS, and BR: conduct of experiments. MC, CPag, MO, SA, and LC: result analysis and interpretation. MC, LM, PM, EC, and LC: manuscript preparation, revisions, and approval. All authors: read and approved the final manuscript.

### Conflict of Interest

The authors declare that the research was conducted in the absence of any commercial or financial relationships that could be construed as a potential conflict of interest.
